# Bakery waste as a sustainable feed resource for growing Awassi lambs: Effects on growth performance, nutrient utilization, carcass traits, meat quality, and health indicators

**DOI:** 10.14202/vetworld.2026.4230-4243

**Published:** 2026-09-24

**Authors:** Belal S. Obeidat, Hadil S. Subih, Linda Alyahya

**Affiliations:** 1 Department of Animal Production, Faculty of Agriculture, Jordan University of Science and Technology, P.O. Box 3030, Irbid 22110, Jordan.; 2 Department of Nutrition and Food Technology, Faculty of Agriculture, Jordan University of Science and Technology, P.O. Box 3030, Irbid 22110, Jordan.; 3 School of Pharmaceutical Sciences, Universiti Sains Malaysia, 11800 Pulau Pinang, Malaysia.

**Keywords:** Awassi lambs, bakery waste, carcass traits, feed efficiency, growth performance, meat quality, nutrient digestibility, sustainable feeding

## Abstract

**Background and Aim:**

Bakery waste (BAWA) represents a potentially economical and sustainable alternative feed resource for ruminant production, particularly in regions where conventional feed ingredients are costly or scarce. This study evaluated the effects of dietary BAWA inclusion on nutrient intake and digestibility, growth performance, nitrogen (N) balance, economic efficiency, carcass characteristics, meat quality, fatty acid (FA) composition, and blood biochemical indicators in growing Awassi lambs.

**Materials and Methods:**

Twenty-four male Awassi lambs (initial body weight, 17.33 ± 0.821 kg; age, 75 ± 1.5 days) were randomly allocated to three dietary treatments (n = 8/group): control (CON; 0 g/kg BAWA), BAWA100 (100 g/kg BAWA), and BAWA200 (200 g/kg BAWA) on a dry matter (DM) basis. The 73-day experiment comprised 10 days of adaptation and 63 days of data collection. Nutrient intake, growth performance, feed efficiency, and feeding costs were determined. Five lambs per group were used for digestibility and N balance assessment. Carcass characteristics, meat quality, FA composition, and serum biochemical indicators were also evaluated.

**Results:**

BAWA100 and BAWA200 increased DM, crude protein (CP), and metabolizable energy intake compared with CON (p < 0.02). BAWA200 increased final body weight, total gain, and average daily gain and improved feed efficiency (p ≤ 0.007). The cost of gain was lower with BAWA200 (p < 0.001). DM and CP digestibility increased with BAWA200, while N retention improved with both BAWA treatments (p < 0.05). Fasting live weight, hot and cold carcass weights, and carcass cut weights were greater with BAWA200 (p ≤ 0.03). Dietary BAWA did not adversely affect meat quality or FA composition. Most serum biochemical indicators remained unaffected, whereas aspartate aminotransferase was numerically lower (p = 0.051) and alanine aminotransferase activities were lower in BAWA-fed lambs (p ≤ 0.05).

**Conclusion:**

Dietary inclusion of BAWA, particularly at 200 g/kg DM, improved growth performance, nutrient utilization, N retention, carcass yield, feed efficiency, and economic outcomes without compromising meat quality, FA composition, or health indicators. BAWA can therefore serve as a practical alternative to conventional concentrate ingredients in growing Awassi lamb diets while supporting more economical and sustainable small-ruminant production.

## INTRODUCTION

Food security depends on sustainable agricultural practices that maintain sufficient, reliable food production across crop and livestock sectors. Global population growth has increased the demand for animal-source foods, including meat, milk, and eggs, placing additional pressure on agricultural resources and production systems. Reducing food loss and waste could substantially improve global food security by increasing resource-use efficiency and expanding the food available for human consumption [1]. Food loss and waste are increasingly important social, economic, and environmental concerns in both developed and developing countries [2]. Approximately one-third of global food production is lost or wasted throughout the food supply chain [3]. In Jordan, food waste is estimated at 93 kg/person annually, representing more than 1 million metric tons and an amount reportedly sufficient to feed approximately 1.5 million people for 1 year [4]. Recycling suitable food byproducts as animal feed is a potential strategy to reduce food waste while improving the sustainability of livestock production.

The scarcity and high cost of conventional feed resources are major constraints on small-ruminant production in arid and semiarid regions, including Jordan. Conventional feeds are expensive, whereas locally available traditional feed resources are often limited and seasonal. Consequently, identifying sustainable and economically viable alternative feed resources is important for maintaining productive small-ruminant systems. Incorporating locally available agri-food byproducts into sheep and goat diets may reduce dependence on conventional feed ingredients while supporting more sustainable feed resource use. Furthermore, integrating agro-industrial byproducts into livestock diets may reduce waste disposal and the associated environmental burden. Ruminants are particularly suited to this approach because they can utilize several agro-industrial residues, including bakery byproducts, and convert their nutrients into high-quality animal-source foods such as meat and milk [5].

In Jordan, limited forage availability and the high cost of supplemental feeds remain important constraints on the profitability of livestock production [6]. Barley is among the most widely used grains for feeding small ruminants in the Middle East and may constitute 500–750 g/kg of dietary dry matter (DM) [7, 8]. Feed represents a substantial proportion of production expenditure, and the livestock sector has become highly dependent on barley, partly because of government subsidies [9]. This dependence increases the importance of identifying alternative feed ingredients that can partially replace conventional grains and other ingredients also used for human food and livestock production. Locally available alternative feed resources could therefore reduce feeding costs, improve resource-use efficiency, and enhance the economic sustainability of small-ruminant production.

Bakery waste (BAWA), a byproduct of bakery production, is characterized by relatively high concentrations of non-fibrous carbohydrates and fat [10]. It comprises bakery leftovers such as bread, cookies, and other confectionery products [11–13] and may include raw, cooked, unsold, or otherwise unsuitable-for-sale products. Because of its high energy content, BAWA has potential as an economical alternative energy source in livestock diets and may partially replace conventional cereal grains [5, 14, 15]. Obeidat [16] reported that including BAWA at 50 and 100 g/kg DM in diets for growing Black goat kids reduced feeding costs without adversely affecting growth performance, health indicators, or meat quality. These findings indicate the potential of BAWA as an alternative feed resource; however, responses may vary according to its inclusion level, nutrient composition, animal species, breed, and overall diet formulation.

Despite the potential benefits of BAWA, important knowledge gaps remain regarding its use in growing Awassi lambs. Previous research has demonstrated the feasibility of relatively low BAWA inclusion levels in growing Black goat kids [16], whereas evidence regarding higher inclusion levels in Awassi lamb diets remains limited. This distinction is important because Awassi sheep are a predominant breed in Jordan and other parts of the Middle East, where shortages and high costs of conventional feeds directly affect production efficiency and profitability. In addition, the variability in the chemical composition of BAWA necessitates evaluation of its effects across multiple nutritional, productive, economic, and health-related outcomes. A comprehensive assessment should therefore extend beyond growth performance to determine whether higher BAWA inclusion influences nutrient intake and digestibility, nitrogen (N) utilization, feed efficiency and economic returns, carcass characteristics, meat quality and fatty acid (FA) composition, and blood biochemical indicators. Such information is needed to establish whether BAWA can partially replace conventional concentrate ingredients without compromising animal performance, product quality, or health.

Therefore, this study aimed to evaluate the effects of including BAWA at 100 and 200 g/kg dietary DM in the diets of growing Awassi lambs compared with a diet without BAWA. Specifically, the study assessed nutrient intake, growth performance, feed efficiency and economic outcomes, nutrient digestibility, N balance, carcass and non-carcass characteristics, meat quality, FA composition, and blood biochemical indicators. The study was designed to determine whether BAWA can serve as a practical and sustainable alternative feed resource for growing Awassi lambs while reducing dependence on conventional feed ingredients and maintaining animal and meat quality outcomes.

## MATERIALS AND METHODS

### Ethical approval

The experimental protocol and all procedures involving animals were reviewed and approved by the Institutional Animal Care and Use Committee of Jordan University of Science and Technology (JUST), Jordan (Protocol No. 16/04/12/216A). All animal procedures were conducted in accordance with the approved institutional protocol and the ARRIVE 2.0 guidelines. Before enrollment, the lambs underwent veterinary examination to confirm their clinical health and suitability for participation in the experiment. The animals were subsequently administered an antiparasitic treatment before allocation to the experimental groups. Throughout the study, the lambs were individually housed under natural ventilation and lighting conditions, provided their assigned diets *ad libitum*, and given unrestricted access to drinking water. Animal handling, blood collection, placement in metabolic cages for the digestibility and N balance experiment, transportation, fasting, and slaughter were performed in accordance with the approved experimental protocol, with appropriate measures taken to minimize unnecessary pain, distress, and discomfort. The animals were monitored throughout the experimental period, and all lambs completed the study without reported illness or mortality. Slaughter was performed humanely at the designated slaughterhouse following the experimental feeding period.

### Study period and location

The study was conducted from October 2025 to January 2026 at the Animal Field of the Research and Training Department, JUST, Irbid, Jordan. The feeding trial lasted 73 days, comprising a 10-day adaptation period followed by 63 days of experimental data collection.

### Study design

A completely randomized design was used. Twenty-four male Awassi lambs, aged 75 ± 1.5 days and with an initial body weight of 17.33 ± 0.821 kg, were stratified by body weight and randomly allocated to three dietary treatment groups (n = 8/group). The treatments consisted of a control diet without BAWA (CON), a diet containing 100 g/kg BAWA (BAWA100), and a diet containing 200 g/kg BAWA (BAWA200), on a DM basis. Each lamb was the experimental unit. The study evaluated nutrient intake, growth performance, feed efficiency, economic outcomes, nutrient digestibility, N balance, carcass characteristics, meat quality, FA composition, and blood biochemical indicators.

### Lambs and experimental diets

Before enrollment, a veterinarian examined all lambs and confirmed they were clinically healthy. They were subsequently administered Ivomec (Merck, Madison, NJ, USA) to control internal parasites and were transferred to the experimental pens. Lambs were individually housed in 1 × 1.5 m pens under natural ventilation and lighting conditions. They were offered their respective diets *ad libitum* twice daily at 08:00 and 15:00 and had unrestricted access to drinking water throughout the study.

The CON, BAWA100, and BAWA200 diets contained 0, 100, and 200 g/kg BAWA on a DM basis, respectively. All diets were formulated according to the NRC requirements for growing lambs [17] and were isonitrogenous, containing approximately 160 g/kg crude protein (CP). Table 1 presents the dietary ingredients, chemical composition of the experimental diets, and chemical composition of BAWA.

BAWA, consisting predominantly of bread with some cookies, was obtained from eateries on the JUST campus. To minimize variation in nutrient composition during the experiment, the quantity required for the entire study was collected as a single batch. The collected BAWA was sun-dried, ground, tested for mycotoxins, and subsequently incorporated into the experimental diets.

Feed refusals were weighed daily, and 10% of the refusals were sampled, dried at 55°C for 48 h, ground to pass through a 1-mm screen, and retained for subsequent analyses. Samples used to determine daily nutrient intake were stored in nylon bags at −20°C until analysis. Lambs were weighed before the morning feeding at the beginning of the experiment and every 2 weeks thereafter. Average daily gain (ADG) was calculated as the difference between final and initial body weights divided by the number of experimental days. Total weight gain and feed efficiency were also determined. Feed efficiency was calculated as total DM intake divided by total body weight gain.

Feed and refusal samples were analyzed according to AOAC procedures [18]. DM was determined by drying samples in a forced-air oven at 100°C for 24 h, CP was determined using the Kjeldahl method, and ether extract (EE) was determined using the Soxtec method. Neutral detergent fiber (NDF) and acid detergent fiber (ADF) were determined using an ANKOM2000 Fiber Analyzer (ANKOM Technology Corporation, Fairport, NY, USA).

### Economic evaluation

Economic efficiency was assessed using diet and body weight gain costs. Diet cost was calculated from the inclusion rate and 2025 price of each dietary ingredient. Cost of gain was calculated by multiplying the feed conversion value by the cost per kilogram of diet. Other production-related costs, including labor, water, electricity, and BAWA processing, were incorporated into the diet and gain cost calculations.

**Table 1:** Ingredients and chemical composition of the diets fed to lambs.

**Item**	**CON**	**BAWA100**	**BAWA200**
Ingredients (g/kg DM)			
Barley grain	490	390	295
Soybean meal	210	210	205
Wheat straw	280	280	280
Bakery waste¹	0	100	200
Salt	10	10	10
Limestone	9	9	9
Vitamin–mineral premix²	1	1	1
Cost (US$/1,000 kg)³	477	443	406
Nutrients			
DM (g/kg)	920	917	913
CP (g/kg)	159	160	159
NDF (g/kg)	324	304	285
ADF (g/kg)	143	138	133
EE (g/kg)	9.5	9.5	9.5
ME (Mcal/kg)⁴	2.31	2.36	2.40

CON = Control diet without bakery waste; BAWA100 = Diet containing 100 g/kg bakery waste; BAWA200 = Diet containing 200 g/kg bakery waste; DM = Dry matter; CP = Crude protein; NDF = Neutral detergent fiber; ADF = Acid detergent fiber; EE = Ether extract; ME = Metabolizable energy.

¹Bakery waste contained 880, 120, 24, 6, 16, and 15 g/kg of DM, CP, NDF, ADF, EE, and sodium, respectively, and 3.2 Mcal/kg of ME. ²Composition per kg: vitamin A, 600,000 IU; vitamin D₃, 200,000 IU; vitamin E, 75 mg; vitamin K₃, 200 mg; vitamin B₁, 100 mg; vitamin B₅, 500 mg; lysine, 0.5%; DL-methionine, 0.15%; manganese oxide, 4,000 mg; ferrous sulfate, 15,000 mg; zinc oxide, 7,000 mg; magnesium oxide, 4,000 mg; potassium iodide, 80 mg; sodium selenite, 150 mg; copper sulfate, 100 mg; cobalt phosphate, 50 mg; and dicalcium phosphate, 10,000 mg. ³Calculated based on ingredient prices in 2025. ⁴Calculated based on the tabular values of NRC [17].

### Digestion and N balance experiment

On day 49 of the feeding period, five lambs were randomly selected from each dietary group (n = 5/group) and individually housed in 1.05 × 0.80 m metabolic cages to evaluate nutrient digestibility and N balance. Lambs were allowed 5 days to adapt to the metabolic cages, followed by a 5-day collection period. Daily feed intake and refusals were recorded throughout the collection period.

Feces were collected and weighed daily, and a 10% subsample was retained for subsequent analysis. Urine was collected simultaneously in plastic containers, weighed, and recorded, and a 5% subsample was retained to determine N balance, including N intake, fecal and urinary N losses, N retention (g/day), and percentage N retention. Each urine collection container contained 50 mL of 6 N HCl to minimize ammonia loss. Retained samples were stored at −20°C until analysis.

At the end of the collection period, fecal and urine samples were composited separately for each lamb. Fecal samples were dried at 50°C to constant weight, ground to pass through a 1-mm screen, and analyzed for DM, NDF, ADF, CP, and EE. Urine samples were analyzed for N content using the Kjeldahl method.

### Slaughtering procedures

At the end of the 63-day data collection period, the lambs were fasted for approximately 18 h, and fasting live body weight was recorded before slaughter. The lambs were transported to the slaughterhouse at approximately 09:00 and humanely slaughtered. Hot carcass weight was recorded immediately after slaughter. Non-carcass components, including the heart, spleen, liver, kidneys, lungs, and trachea, were removed and weighed.

Carcasses were chilled at 4°C for 24 h, after which cold carcass weight and other carcass measurements were recorded. Dressing percentage was calculated as cold carcass weight divided by fasting live body weight × 100. Fat depth (C), eye muscle depth (B), eye muscle width (A), tissue depth (GR), rib fat depth (J), and eye muscle area were subsequently measured. The carcasses were divided into four major cuts: loin, shoulder, leg, and rack. The *longissimus dorsi* muscle was removed from the loin for meat quality analyses, vacuum-packaged in plastic bags, and stored at −20°C for 14 days until analysis.

### Meat quality measurements

After frozen storage, vacuum-packaged *longissimus dorsi* muscle samples were thawed overnight at 4°C. Meat pH, shear force, cooking loss (CL), water-holding capacity (WHC), and CIELAB color coordinates (L*, a*, and b*) were determined in triplicate. Samples were cut to standardized thicknesses according to the requirements of each analysis.

For pH determination, 2 g of raw meat was homogenized in 10 mL of neutralized 5 mM iodoacetate reagent, and the homogenate pH was measured using a pH/temperature tester (Model 35634-40, Eurotech Instruments, Malaysia). Meat color was measured using the CIELAB system with a Chroma Meter CR-410 (Konica Minolta) on 15-mm-thick slices placed on polystyrene trays, covered with a porous sheet, and maintained at 4°C for 2 h before measurement.

CL was determined using 25-mm-thick meat slices. Samples were weighed before cooking, sealed in plastic bags, and cooked *sous vide* at 75°C for 90 min. Samples were reweighed after cooking to determine weight loss. Cooked samples were subsequently refrigerated at 4°C overnight for shear force determination and divided into six 1-cm³ cores. Maximum force (kg) required to shear the cooked samples was determined using a Warner–Bratzler shear blade with a triangular slot and sharp edge (GR Manufacturing Co., Manhattan, KS, USA) and a Salter Model 235.

WHC was determined using the method of Grau and Hamm [19]. Briefly, 5 g of raw meat was subjected to a 2,500-g load between quartz plates and filter papers for 5 min. The sample was subsequently removed and weighed. WHC was calculated as follows:

WHC (%) = [(initial weight − final weight)/initial weight] × 100.

### FA analysis

The FA composition of meat was analyzed at the Military Quality Control Laboratories, Amman, Jordan, an accredited analytical laboratory operating under standardized quality-assurance procedures. FAs were converted to FA methyl esters (FAMEs) by transesterification as described by Jawasreh *et al.* [20].

FAMEs were separated and quantified using a Shimadzu GC-2010 Plus gas chromatograph (GC-MS-TQ8040 NX GC-MS/MS, Kyoto, Japan) equipped with a fused-silica capillary DB-WAX column (SP-2380, 100 m × 0.25 mm; Agilent Technologies, USA). Helium was used as the carrier gas, and detection was performed using a flame-ionization detector.

### Blood biochemical analysis

Blood samples were collected from the jugular vein of each lamb on days 1, 30, and 60 before the morning feeding at 08:00. Samples were collected into Vacutainer tubes without anticoagulant for serum separation. Blood samples were centrifuged at 3000 rpm for 30 min, and the separated serum was transferred into tubes and stored at −20°C until analysis.

Serum glucose, triglycerides, cholesterol, creatinine, urea N, low-density lipoprotein (LDL), and high-density lipoprotein (HDL) concentrations were determined. Liver-associated biochemical indicators included aspartate aminotransferase (AST), alkaline phosphatase (ALP), and alanine aminotransferase (ALT). Analyses were performed using commercial kits (Biolabo S.A.S., Maizy, France) according to the manufacturer's instructions, and absorbance was measured using a JENWAY 6105 UV/Vis spectrophotometer (Jenway Ltd., Felsted, Dunmow, Essex, UK).

### Statistical analysis

Data were analyzed using the MIXED procedure of SAS (version 8.1, SAS Institute Inc., Cary, NC, USA). Diet (CON, BAWA100, and BAWA200) was included as a fixed effect, and lamb was included as a random effect. The individual lamb was considered the experimental unit.

Serum biochemical data were analyzed using repeated-measures analysis of variance, with diet, sampling day, and diet × day interaction included in the model. The covariance structure was selected from compound symmetry, first-order autoregressive, and unstructured covariance structures according to the Schwarz Bayesian criterion. Because no significant diet × day interaction was detected, only the main effects were reported.

When a fixed effect was significant (p ≤ 0.05), pairwise *t*-tests were performed to compare least-squares means. Statistical significance was established at p ≤ 0.05.

## RESULTS

All lambs completed the study without illness or mortality. The BAWA200 diet had the lowest feed cost, followed by the BAWA100 and CON diets (Table 1).

Table 2 presents the effects of BAWA inclusion on nutrient intake and growth performance. Lambs fed BAWA100 and BAWA200 had greater DM, CP, and metabolizable energy (ME) intakes than those fed CON (p < 0.02). NDF intake also differed among treatments (p = 0.002), with the lowest value observed in the BAWA200 group. ADF and EE intakes did not differ among dietary treatments (p > 0.05). The BAWA200 group had higher final body weight, total weight gain, and ADG than the BAWA100 and CON groups (p ≤ 0.005). Feed efficiency also improved in the BAWA200 group (p = 0.007), and the cost of gain was lower than in the BAWA100 and CON groups (p = 0.001).

**Table 2:** Effects of bakery waste inclusion on nutrient intake and growth performance of lambs.

**Item**	**CON (n = 8)**	**BAWA100 (n = 8)**	**BAWA200 (n = 8)**	**SEM**	**p-value**
**Nutrient intake**					
DM (g/day)	1098ᵃ	1158ᵇ	1142ᵇ	19.01	0.019
CP (g/day)	175ᵃ	185ᵇ	182ᵇ	3.0	0.011
NDF (g/day)	356ᵃ	353ᵇ	326ᵇ	5.5	0.002
ADF (g/day)	157	160	152	5.1	0.341
EE (g/day)	10	11	11	0.3	0.241
ME (Mcal/day)	2.54ᵃ	2.73ᵇ	2.74ᵇ	0.046	0.001
Initial weight (kg)	17.06	16.77	18.17	0.821	0.136
Final weight (kg)	28.03ᵃ	28.80ᵃ	33.63ᵇ	1.431	0.003
Total gain (kg)	10.97ᵃ	12.03ᵃ	15.46ᵇ	1.176	0.005
Average daily gain (g/day)	182.86ᵃ	200.48ᵃ	257.62ᵇ	19.603	0.005
Feed efficiency	6.14ᵇ	6.00ᵇ	4.53ᵃ	0.468	0.007
Cost of gain (US$/kg gain)	2.93ᵇ	2.64ᵇ	1.84ᵃ	0.208	0.001

CON = Control diet without bakery waste; BAWA100 = Diet containing 100 g/kg bakery waste; BAWA200 = Diet containing 200 g/kg bakery waste; DM = Dry matter; CP = Crude protein; NDF = Neutral detergent fiber; ADF = Acid detergent fiber; EE = Ether extract; ME = Metabolizable energy; SEM = Standard error of the mean. ᵃ^,^ ᵇValues within a row with different superscript letters differ significantly (p ≤ 0.05).

Table 3 presents the effects of BAWA inclusion on *in vivo* nutrient digestibility and N balance. DM digestibility was greater in the BAWA200 group than in the CON group (p = 0.010), whereas BAWA100 was intermediate. CP digestibility increased across treatments and was highest in the BAWA200 group (p = 0.002). NDF, ADF, and EE digestibility did not differ among treatments (p > 0.05). N intake was greater in the BAWA100 and BAWA200 groups than in the CON group (p = 0.046). Fecal and urinary N excretion did not differ significantly among treatments (p > 0.05). However, N retained and percentage N retention were greater in the BAWA100 and BAWA200 groups than in the CON group (p = 0.002).

Table 4 presents the effects of BAWA inclusion on carcass characteristics, non-carcass components, carcass cut weights, and loin tissue composition. Fasting live weight, hot carcass weight, cold carcass weight, and total carcass cut weight were greater in the BAWA200 group than in the BAWA100 and CON groups (p ≤ 0.017). Dressing percentage, kidney fat, fat-tail weight, non-carcass component weight, loin weight, subcutaneous fat, total fat, total lean, total bone, meat-to-bone ratio, and meat-to-fat ratio did not differ among treatments (p > 0.05). Mesenteric fat and intermuscular fat were lower in the BAWA100 group than in the BAWA200 group, whereas the CON group was intermediate.

**Table 3:** Effects of bakery waste inclusion on in vivo nutrient digestibility and nitrogen balance in lambs.

**Item**	**CON (n = 5)**	**BAWA100 (n = 5)**	**BAWA200 (n = 5)**	**SEM**	**p-value**
Digestibility coefficients					
DM	82.8ᵃ	85.3ᵃᵇ	87.3ᵇ	0.76	0.010
CP	80.8ᵃ	82.9ᵇ	85.6ᶜ	0.79	0.002
NDF	64.9	65.3	68.5	1.23	0.141
ADF	57.0	56.3	58.0	2.31	0.877
EE	81.7	82.1	84.5	1.98	0.573
N balance					
N intake (g/day)	22.76ᵃ	24.52ᵇ	24.94ᵇ	0.590	0.046
N in feces (g/day)	4.36	4.19	3.59	0.509	0.067
N in urine (g/day)	9.73	8.60	8.66	0.345	0.080
N retained (g/day)	8.67ᵃ	11.33ᵇ	12.69ᵇ	0.920	0.002
N retention (g/100 g)	37.49ᵃ	47.71ᵇ	50.87ᵇ	3.160	0.002

CON = Control diet without bakery waste; BAWA100 = Diet containing 100 g/kg bakery waste; BAWA200 = Diet containing 200 g/kg bakery waste; DM = Dry matter; CP = Crude protein; NDF = Neutral detergent fiber; ADF = Acid detergent fiber; EE = Ether extract; N = Nitrogen; SEM = Standard error of the mean.

ᵃ^–^ᶜValues within a row with different superscript letters differ significantly (p ≤ 0.05).

**Table 4:** Effects of bakery waste inclusion on carcass characteristics, non-carcass components, carcass cut weights, and loin tissue composition of lambs.

**Item**	**CON (n = 8)**	**BAWA100 (n = 8)**	**BAWA200 (n = 8)**	**SEM**	**p-value**
Fasting live weight (kg)	27.34ᵃ	27.51ᵃ	32.43ᵇ	1.492	0.006
Hot carcass weight (kg)	13.91ᵃ	13.69ᵃ	16.49ᵇ	0.856	0.001
Cold carcass weight (kg)	13.34ᵃ	13.06ᵃ	15.94ᵇ	0.893	0.011
Dressing percentage	48.54	47.20	49.05	1.046	0.222
Mesenteric fat (g)	96.43ᵃᵇ	81.43ᵃ	113.57ᵇ	10.645	0.030
Kidney fat (g)	170.71	132.86	155.14	29.567	0.457
Fat tail (g)	864.29	595.36	941.43	206.18	0.246
Non-carcass components (kg)¹	1.31	1.26	1.47	0.081	0.084
Carcass cut weights (kg)²	11.86ᵃ	11.83ᵃ	14.38ᵇ	0.882	0.017
Loin weight (g)	492.2	476.4	599.0	55.32	0.088
Intermuscular fat (g)	45.6ᵃᵇ	33.3ᵃ	64.0ᵇ	9.17	0.015
Subcutaneous fat (g)	41.0	33.2	39.5	8.13	0.609
Total fat (g/100 g)	17.2	13.2	17.1	2.14	0.135
Total lean (g/100 g)	53.8	55.7	57.6	2.31	0.302
Total bone (g/100 g)	21.3	24.7	22.2	2.10	0.271
Meat-to-bone ratio	2.54	2.46	2.61	0.253	0.841
Meat-to-fat ratio	3.24	6.18	3.67	1.650	0.193

CON = Control diet without bakery waste; BAWA100 = Diet containing 100 g/kg bakery waste; BAWA200 = Diet containing 200 g/kg bakery waste; SEM = Standard error of the mean. ¹Non-carcass components included heart, liver, spleen, kidneys, lungs, and trachea. ²Carcass cuts included shoulder, racks, loins, and legs. ᵃ^,^ᵇValues within a row with different superscript letters differ significantly (p ≤ 0.05).

BAWA inclusion did not affect most carcass linear measurements (Table 5). Tissue depth was greater in lambs fed BAWA200 than in those fed BAWA100 and CON (p = 0.0208). Rib fat depth also differed among treatments (p = 0.0006), with the greatest value in BAWA200, an intermediate value in CON, and the lowest value in BAWA100. Leg fat depth, eye muscle area, eye muscle width, eye muscle depth, fat depth, and shoulder fat depth did not differ among treatments (p > 0.05).

**Table 5:** Effects of bakery waste inclusion on carcass linear measurements in lambs

**Item**	**CON (n = 8)**	**BAWA100 (n = 8)**	**BAWA200 (n = 8)**	**SEM**	**p-value**
Leg fat depth, L3 (mm)	2.08	1.86	2.86	0.602	0.2502
Tissue depth, GR (mm)	8.86ᵃ	8.29ᵃ	11.43ᵇ	1.039	0.0208
Rib fat depth, J (mm)	2.93ᵇ	2.07ᵃ	3.71ᶜ	0.320	0.0006
Eye muscle area (cm²)	6.67	7.63	8.21	0.694	0.1175
Eye muscle width, A (mm)	47.43	47.58	50.08	2.074	0.3823
Eye muscle depth, B (mm)	21.36	20.86	22.50	1.231	0.4176
Fat depth, C (mm)	1.29	1.58	1.43	0.286	0.6132
Shoulder fat depth, S2 (mm)	1.00	1.14	1.00	0.101	0.321

CON = Control diet without bakery waste; BAWA100 = Diet containing 100 g/kg bakery waste; BAWA200 = Diet containing 200 g/kg bakery waste; SEM = Standard error of the mean. ᵃ^–^ᶜValues within a row with different superscript letters differ significantly (p ≤ 0.05).

Dietary treatments did not differ significantly in meat pH, cooking loss, water-holding capacity, shear force, or color coordinates (Table 6; p > 0.05).

**Table 6:** Effects of bakery waste inclusion on physicochemical properties of the longissimus dorsi muscle in lambs

**Item**	**CON (n = 8)**	**BAWA100 (n = 8)**	**BAWA200 (n = 8)**	**SE**	**p-value**
pH¹	5.85	5.91	5.88	0.037	0.284
Cooking loss (g/100 g)	40.70	44.76	43.43	3.770	0.561
Water-holding capacity (g/100 g)	32.42	31.05	31.49	2.056	0.796
Shear force (kg/cm²)	7.46	7.59	6.45	1.030	0.497
Color coordinates					
L* (lightness)	40.60	43.41	42.12	2.041	0.411
a* (redness)	16.75	16.03	17.26	0.593	0.106
b* (yellowness)	11.62	11.47	12.33	0.563	0.300

CON = Control diet without bakery waste; BAWA100 = Diet containing 100 g/kg bakery waste; BAWA200 = Diet containing 200 g/kg bakery waste; SE = Standard error. ¹pH was measured after thawing.

BAWA inclusion did not significantly affect blood urea nitrogen (BUN), glucose, cholesterol, HDL, LDL, triglycerides, creatinine, or ALP (Table 7; p > 0.05). ALT activity was lower in the BAWA100 and BAWA200 groups than in the CON group (p = 0.001). AST showed a numerical reduction in both BAWA groups, but the overall treatment effect did not reach the predefined significance threshold (p = 0.051).

**Table 7:** Effects of bakery waste inclusion on blood biochemical indicators in lambs

**Item**	**CON (n = 8)**	**BAWA100 (n = 8)**	**BAWA200 (n = 8)**	**SEM**	**p-value**
Blood urea nitrogen (mg/dL)	22.79	25.88	26.41	1.728	0.230
Glucose (mg/dL)	65.25	72.06	82.25	6.168	0.171
Cholesterol (mg/dL)	62.50	61.56	67.81	4.290	0.549
HDL (mg/dL)	26.81	26.94	28.75	1.216	0.465
LDL (mg/dL)	32.66	27.69	30.99	3.953	0.670
Triglycerides (mg/dL)	15.27	12.31	14.15	1.932	0.559
Creatinine (mg/dL)	0.76	0.74	0.78	0.017	0.381
AST (IU/L)	50.08	32.19	33.94	5.323	0.051
ALT (IU/L)	13.94ᵇ	7.31ᵃ	8.06ᵃ	1.173	0.001
ALP (IU/L)	26.69	31.31	30.81	3.00	0.500

CON = Control diet without bakery waste; BAWA100 = Diet containing 100 g/kg bakery waste; BAWA200 = Diet containing 200 g/kg bakery waste; HDL = High-density lipoprotein; LDL = Low-density lipoprotein; AST = Aspartate aminotransferase; ALT = Alanine aminotransferase; ALP = Alkaline phosphatase; SEM = Standard error of the mean.

ᵃ^,b^Values within a row with different superscript letters differ significantly (p ≤ 0.05).

The FA profile of the *longissimus dorsi* muscle did not differ significantly among dietary treatments (Table 8; p > 0.05). Total saturated, monounsaturated, and polyunsaturated FA proportions were also comparable among the CON, BAWA100, and BAWA200 groups.

**Table 8:** Effects of bakery waste inclusion on the meat fatty acid profile of lambs

**FA (%)**	**CON (n = 8)**	**BAWA100 (n = 8)**	**BAWA200 (n = 8)**	**SEM**	**p-value**
Saturated FA					
C4:0	0.016	0.020	0.016	0.0035	0.444
C8:0	0.019	0.016	0.019	0.0024	0.340
C10:0	0.208	0.211	0.219	0.0095	0.508
C12:0	0.316	0.220	0.237	0.0840	0.490
C13:0	0.113	0.108	0.126	0.0404	0.900
C14:0	3.201	3.407	3.131	0.4682	0.831
C15:0	0.426	0.407	0.380	0.0704	0.811
C16:0	25.440	25.695	26.170	0.3157	0.098
C17:0	1.342	1.255	1.090	0.1368	0.210
C18:0	15.163	15.215	14.635	0.4462	0.382
C20:0	0.077	0.082	0.072	0.0066	0.317
C24:0	0.035	0.038	0.045	0.0043	0.109
Total saturated FA	46.36	46.67	46.14	0.859	0.825
Monounsaturated FA					
C14:1	0.347	0.341	0.324	0.0349	0.789
C15:1	0.128	0.132	0.143	0.0173	0.667
C16:1	2.724	2.573	2.398	0.1858	0.248
C17:1	1.133	1.289	0.961	0.1720	0.535
C18:1 cis + trans (n-9)	44.750	44.064	45.115	0.6418	0.283
C20:1	0.128	0.132	0.125	0.0076	0.641
C21:1	0.018	0.015	0.018	0.0019	0.259
Total monounsaturated FA	49.23	48.38	49.09	0.663	0.4164
Polyunsaturated FA					
C18:2 cis-9, cis-12 (n-6)	2.996	2.887	2.905	0.0868	0.423
C18:3 (n-6)	0.200	0.202	0.193	0.0249	0.931
C18:3 (n-3)	0.373	0.436	0.456	0.0713	0.502
C20:2	0.054	0.047	0.046	0.0067	0.457
C20:3 (n-6)	0.159	0.223	0.221	0.0513	0.390
Total polyunsaturated FA	3.78	3.78	3.69	0.152	0.7720

CON = Control diet without bakery waste; BAWA100 = Diet containing 100 g/kg bakery waste; BAWA200 = Diet containing 200 g/kg bakery waste; FA = Fatty acid; SEM = Standard error of the mean.

## DISCUSSION

### BAWA as an alternative feed resource

The increasing cost of conventional feeds has intensified interest in identifying alternative feed resources that can reduce production costs while supporting livestock productivity and environmental sustainability [21]. In the present study, partial replacement of conventional concentrate ingredients with BAWA reduced dietary costs and, particularly at the 200 g/kg DM inclusion level, improved growth performance and feed efficiency without adversely affecting meat quality or most measured blood biochemical indicators. These findings support the potential use of BAWA as a practical feed resource in small-ruminant production systems where conventional feed ingredients are costly or limited.

The chemical composition of BAWA was sufficiently compatible with the conventional ingredients used in the experimental diets to maintain comparable concentrations of CP, EE, and ME across treatments, averaging 159.3 g/kg, 9.50 g/kg, and 2.36 Mcal/kg, respectively (Table 1). Nevertheless, the chemical composition of BAWA can vary considerably among sources [10, 11, 14]. This variation may reflect differences in the type and proportion of bakery products included, fat content, processing conditions, and material source [10, 11, 13]. This variability can complicate diet formulation and comparisons among studies. Therefore, chemical characterization of BAWA before dietary inclusion remains important to ensure appropriate nutrient formulation and consistency of feeding outcomes [11].

### Feed cost, nutrient intake, and growth performance

Increasing BAWA inclusion progressively reduced diet cost (Table 1). Compared with the CON diet, inclusion of BAWA at 100 and 200 g/kg DM reduced diet cost by US$34/1,000 kg (7.1%) and US$71/1,000 kg (14.9%), respectively. This finding is consistent with previous studies reporting economic benefits from replacing conventional feed ingredients with bakery or other alternative feed resources [8, 22, 23]. The lower cost observed in the present study is attributable primarily to partial replacement of more expensive conventional ingredients, particularly barley grain, and is consistent with reports that alternative feed resources can reduce feeding expenditure [15, 16, 24, 25].

Lambs fed the BAWA diets had greater DM intake than those fed the CON diet, which was accompanied by greater CP and ME intake (Table 2). ME intake averaged approximately 2.7 Mcal/day in the BAWA groups compared with 2.5 Mcal/day in the CON group. These findings indicate that inclusion of BAWA at 100 or 200 g/kg DM did not reduce diet acceptance under the conditions of the present study. Obeidat [16] similarly reported increased DM intake in growing Black goat kids fed 100 g/kg BAWA compared with controls. Haddad and Ereifej [8] also found that replacement of barley grain with bread byproduct at up to 200 g/kg of diet did not reduce DM intake in growing kids. Similarly, França *et al.* [11] reported that BAWA inclusion at 250, 500, 750, and 1,000 g/kg dietary DM did not significantly alter nutrient intake in sheep.

Growth performance was greatest in lambs fed BAWA200. This group had higher final body weight, total gain, and ADG than the BAWA100 and CON groups, while feed efficiency and cost of gain also improved. The greater growth response observed with BAWA200 coincided with greater nutrient and energy intake and therefore may, at least in part, reflect the increased availability of dietary nutrients. Almahdawi and Altalib [5] similarly reported higher daily and total weight gain in lambs fed diets containing dried bread residues. Salama *et al.* [26] also observed favorable effects on live weight gain when lambs received bakery byproducts at 125–500 g/kg DM.

However, previous studies have not reported uniform growth responses. Hindiyeh *et al.* [22] found reduced growth performance in Awassi lambs when BAWA replaced barley grain at 100, 200, and 300 g/kg dietary DM, whereas Afzalzadeh *et al.* [24] reported no significant differences in final body weight, daily gain, or total gain in Zandi lambs receiving 0–250 g/kg BAWA. Differences among studies may reflect variation in BAWA composition, dietary formulation, inclusion level, animal breed, age, and processing method. In the present study, the improved productive response at 200 g/kg DM was accompanied by lower feed cost and lower cost per kilogram of gain, indicating a favorable economic effect under the experimental conditions.

### Nutrient digestibility and N utilization

Lambs fed the BAWA200 diet had greater DM and CP digestibility than those fed the CON diet, whereas NDF, ADF, and EE digestibility did not differ among dietary treatments. These findings indicate that including BAWA at the tested levels did not impair overall nutrient utilization and that the higher inclusion level was associated with improved DM and CP digestibility. Differences in nutrient intake among treatments may have contributed to these responses.

Mahmoud [13] reported improved CP digestibility and digestible CP in growing lambs fed diets containing 300 and 600 g/kg DM bakery byproducts. Salama *et al.* [26] likewise reported increased DM digestibility in lambs receiving diets containing bakery byproducts. In contrast, Obeidat [16] found no change in nutrient digestibility when BAWA replaced barley grain at 50 or 100 g/kg DM. França *et al.* [11] also reported that BAWA inclusion did not affect DM digestibility in sheep. Similarly, Obeidat *et al.* [27] found no effect of bread byproduct inclusion at 100, 150, or 200 g/kg on nutrient digestibility in Awassi ewes, and Haddad and Ereifej [8] reported no effect of increasing bread byproduct inclusion on DM, organic matter, or NDF digestibility in goat kids. Collectively, these differing results suggest that digestibility responses to bakery byproducts depend on byproduct composition and overall dietary formulation.

BAWA inclusion also influenced N utilization. Lambs fed BAWA100 and BAWA200 had greater N intake, N retained, and percentage N retention than those fed the CON diet, whereas fecal and urinary N excretion did not differ significantly among treatments. The greater N retention may be related to the higher CP intake and, particularly in BAWA200, greater CP digestibility. França *et al.* [11] reported that BAWA could be included in sheep diets at 250–1,000 g/kg DM without adversely affecting N balance, whereas Obeidat [16] found no differences in N intake or N balance when BAWA was included at 50 or 100 g/kg DM. The present findings therefore indicate that, under the current dietary conditions, BAWA inclusion did not compromise N utilization and was associated with greater N retention.

### Carcass characteristics and fat deposition

The greater fasting live weight, hot carcass weight, cold carcass weight, and carcass cut weight observed in the BAWA200 group were consistent with the greater growth performance of these lambs. Improved nutrient intake and digestibility may have contributed to the increased carcass weights, although the present study was not designed to establish a direct mechanistic relationship. Mesenteric and intermuscular fat weights were also greater in BAWA200 than in BAWA100, which may reflect differences in energy intake and nutrient partitioning between the groups.

Obeidat [16] reported that inclusion of BAWA at 50 and 100 g/kg in diets of Black goat kids increased loin cut weight and total lean muscle weight but did not substantially alter other carcass characteristics. In finishing beef steers, Milton and Brandt [28] reported similar hot carcass weights and dressing percentages when dried bakery products replaced corn at 15% and 30% of the diet, although kidney, pelvic, and heart fat increased linearly. Guiroy *et al.* [29] similarly found no differences in dressing percentage or fat depth between corn-based and bread-byproduct-based diets in growing-finishing beef cattle. Obeidat *et al.* [27] also reported no differences in carcass weight, internal fat weight, or fat-tail weight in lambs receiving BAWA at 60–250 g/kg DM. These comparisons further indicate that carcass responses to bakery byproducts may depend on inclusion rate, dietary energy supply, species, breed, and overall feeding strategy.

Among the carcass linear measurements evaluated, only tissue depth and rib fat depth differed among treatments (Table 5). Tissue depth was greater in lambs fed BAWA200 than in those fed BAWA100 or CON, whereas rib fat depth was greatest in BAWA200, intermediate in CON, and lowest in BAWA100. These differences are compatible with the greater growth and energy intake observed in BAWA200. Diet, breed, sex, age, and energy intake are recognized factors influencing carcass fat deposition [30]. Obeidat [16] likewise observed greater rib fat depth in goat kids fed 100 g/kg BAWA than in those receiving 50 or 0 g/kg BAWA. In contrast, Milton and Brandt [28] reported only a nonsignificant increase in 12^th^-rib fat thickness when bakery products were included in diets for finishing beef steers.

### Meat quality

BAWA inclusion did not significantly affect pH, CL, WHC, shear force, or meat color coordinates (Table 6). Thus, the improvements in growth and carcass weight observed with BAWA200 were not accompanied by detectable adverse changes in the measured physicochemical characteristics of the *longissimus dorsi* muscle.

This finding agrees with Obeidat [16], who reported no adverse effect of BAWA inclusion on meat quality characteristics in growing goat kids. Guiroy *et al.* [29] similarly found that meat palatability traits were not altered when beef steers were fed a bread-byproduct-based diet. Grossi *et al.* [31] also reported no unfavorable effect of bakery-derived feed ingredients on carcass-related outcomes in beef cattle. Together, these findings suggest that moderate inclusion of bakery byproducts can be compatible with maintaining desirable meat quality charac-teristics, although responses may differ according to diet composition and animal production system.

### Blood biochemical indicators

Most measured serum biochemical indicators were unaffected by BAWA inclusion, including BUN, glucose, cholesterol, HDL, LDL, triglycerides, creatinine, and ALP. ALT activity was significantly lower in lambs fed BAWA100 and BAWA200 than in those fed CON, whereas AST showed a numerical reduction but did not reach the predefined significance threshold (p = 0.051; Table 7). 

The lower ALT activity observed in the BAWA groups indicates a dietary effect on this biochemical indicator; however, this finding should be interpreted cautiously because serum enzyme activity alone does not establish improved hepatic health or reduced hepatocellular stress. Mahmoud [13] reported that inclusion of bakery byproducts at 30% and 60% of dietary DM did not alter AST or ALT activity in lambs. Kaltenegger *et al.* [32] likewise found no effect of bakery byproduct inclusion as a grain replacement on AST activity in dairy cows, and Obeidat [16] reported no change in liver-associated enzymes in growing goat kids fed BAWA. Further studies are needed to confirm whether the significantly lower ALT and the numerical reduction in AST are related to BAWA inclusion in growing lamb diets.

### Meat FA profile

Dietary BAWA did not significantly affect individual FAs or the total proportions of saturated, mono-unsaturated, and polyunsaturated FAs in lamb meat (Table 8). This finding indicates that replacing part of the conventional concentrate ingredients with BAWA at 100 or 200 g/kg DM did not materially alter the measured FA profile of the *longissimus dorsi* muscle under the conditions of the present study.

Diet is an important determinant of meat FA composition [33,34], and the lipid sources present in bakery byproducts may vary depending on whether the original products contain dairy fats, vegetable oils, or other fat sources [35]. Nevertheless, in ruminants, extensive ruminal biohydrogenation of dietary unsaturated FAs can reduce the direct relationship between dietary and tissue FA profiles [36]. This process, together with endogenous FA synthesis, desaturation, and elongation, contributes substantially to the final FA composition of ruminant tissues [37–39]. When dietary lipid supply is moderate and broadly comparable among treatments, endogenous metabolism and ruminal biohydrogenation may attenuate diet-related differences in tissue FAs [40, 41].

The absence of significant differences in the meat FA profile is therefore consistent with the similar EE concentrations of the experimental diets and the lack of major differences in total dietary lipid supply. Importantly, the present data indicate that BAWA could partially replace conventional concentrate ingredients without compromising the measured FA composition of lamb meat. However, because BAWA can vary considerably in nutrient and ingredient composition, appropriate quality control remains essential before its use in animal diets. Particular attention should be given to variability in fat composition, salt concentration, and potential mycotoxin contamination.

## CONCLUSION

The present study demonstrated that BAWA can be incorporated into diets of growing Awassi lambs at 100 and 200 g/kg DM without adverse effects on animal health, meat physicochemical characteristics, or meat FA composition. Among the tested inclusion levels, BAWA200 produced the most favorable overall response. Lambs fed BAWA200 had greater final body weight, total weight gain, ADG, DM and CP digestibility, and carcass weights, together with improved feed efficiency. Both BAWA100 and BAWA200 increased N retention compared with the CON diet, while BAWA inclusion reduced diet cost and cost of gain. Most measured blood biochemical indicators remained unchanged, whereas ALT activity was lower in the BAWA-fed groups.

From a practical perspective, these findings indicate that BAWA can partially replace conventional concentrate ingredients, particularly barley grain, in diets of growing Awassi lambs and may provide an economically attractive feeding strategy in regions where conventional feed resources are expensive or limited. The reduction in feed cost, together with the improved productive performance observed with BAWA200, supports BAWA's potential to improve the economic sustainability of small-ruminant production while providing a productive use for bakery-derived food waste.

A major strength of this study was the comprehensive evaluation of BAWA across nutrient intake, growth performance, feed efficiency, economic outcomes, nutrient digestibility, N balance, carcass characteristics, meat quality, FA composition, and blood biochemical indicators within the same controlled feeding experiment. Nevertheless, several limitations should be considered. The study included a relatively small number of male Awassi lambs and evaluated only two BAWA inclusion levels during a 73-day feeding period. In addition, BAWA was obtained as a single batch, although its chemical composition may vary substantially by source and production period. The study also did not directly evaluate rumen fermentation, microbial populations, long-term metabolic responses, or the mechanisms underlying the observed differences in nutrient utilization and blood biochemical indicators.

Future studies should evaluate additional BAWA inclusion levels in larger and more diverse animal populations and over longer production periods. Research should also assess batch-to-batch variation in BAWA composition, establish practical quality control criteria for nutrient composition, salt content, and mycotoxin contamination, and investigate rumen fermentation, microbial responses, nutrient metabolism, and longer-term health outcomes. Economic assessments under commercial farm conditions would further clarify the practical applicability and cost-effectiveness of this feeding strategy.

Overall, BAWA inclusion at up to 200 g/kg DM appears to be a promising alternative feeding approach for growing Awassi lambs. Under the conditions of this study, BAWA200 provided the most favorable balance among growth performance, nutrient utilization, carcass productivity, feed efficiency, and economic outcomes without compromising the measured meat quality, FA, or health-related indicators. These findings support the potential integration of appropriately characterized BAWA into sustainable small-ruminant feeding systems.

## DATA AVAILABILITY

The data supporting the findings of this study are presented within the article.

## GENERATIVE AI DECLARATION

The authors declare that no generative artificial intelligence (AI) tools were used in the preparation, writing, editing, or revision of this manuscript.

## AUTHORS’ CONTRIBUTIONS

BSO and HSS: Conceptualization, methodology, investigation, supervision, interpretation of results, and writing – original draft. LA: Interpretation of results and writing – review and editing. All authors have read and approved the final manuscript.
